# Molecular Mechanisms and Therapeutic Implications of Human Pericyte-like Adipose-Derived Mesenchymal Stem Cells in an In Vitro Model of Diabetic Retinopathy

**DOI:** 10.3390/ijms25031774

**Published:** 2024-02-01

**Authors:** Aleksandra Agafonova, Alessia Cosentino, Ivana Roberta Romano, Giovanni Giurdanella, Floriana D’Angeli, Rosario Giuffrida, Debora Lo Furno, Carmelina Daniela Anfuso, Giuliana Mannino, Gabriella Lupo

**Affiliations:** 1 Department of Biomedical and Biotechnological Sciences, School of Medicine, University of Catania, 95123 Catania, Italy; aleksandraaagafonova@gmail.com (A.A.); alessia1993@hotmail.it (A.C.); ivanarobertaromano@yahoo.it (I.R.R.); giuffros@unict.it (R.G.); gabriella.lupo@unict.it (G.L.); 2 Faculty of Medicine and Surgery, University of Enna “Kore”, 94100 Enna, Italy; giovanni.giurdanella@unikore.it; 3 Department of Human Sciences and Quality of Life Promotion, San Raffaele Roma Open University, 00166 Rome, Italy; floriana.dangeli@uniroma5.it; 4 Department of Chemical, Biological, Pharmaceutical and Environmental Sciences, University of Messina, 98122 Messina, Italy; giuliana.mannino@unime.it

**Keywords:** adipose mesenchymal stem cells, pericyte-like differentiation, human retinal endothelial cells, blood–retinal barrier, diabetic retinopathy, hyperglycemia, cytosolic phospholipase A2, inflammation, vascular endothelial growth factor, cell-based therapy

## Abstract

The blood–retinal barrier (BRB) is strongly compromised in diabetic retinopathy (DR) due to the detachment of pericytes (PCs) from retinal microvessels, resulting in increased permeability and impairment of the BRB. Western blots, immunofluorescence and ELISA were performed on adipose mesenchymal stem cells (ASCs) and pericyte-like (P)-ASCs by co-cultured human retinal endothelial cells (HRECs) under hyperglycemic conditions (HG), as a model of DR. Our results demonstrated that: (a) platelet-derived growth factor receptor (PDGFR) and its activated form were more highly expressed in monocultured P-ASCs than in ASCs, and this expression increased when co-cultured with HRECs under high glucose conditions (HG); (b) the transcription factor Nrf2 was more expressed in the cytoplasmic fraction of ASCs and in the P-ASC nuclear fraction, under normal glucose and, even more, under HG conditions; (c) cytosolic phospholipase A_2_ activity and prostaglandin E2 release, stimulated by HG, were significantly reduced in P-ASCs co-cultured with HRECs; (d) HO-1 protein content was significantly higher in HG-P-ASCs/HRECs than P-ASCs/HRECs; and (e) VEGF-A levels in media from HG-co-cultures were reduced in P-ASCs/HRECs with respect to ASCs/HRECs. The data obtained highlighted the potential of autologous differentiated ASCs in future clinical applications based on cell therapy to counteract the damage induced by DR.

## 1. Introduction

Diabetic retinopathy (DR) is characterized by morphological and metabolic alterations of the microvascular cells forming the blood–retinal barrier (BRB). In the retinae of diabetic patients, pericyte (PC) loss and the formation of microaneurysms are early hallmarks of diabetic retinopathy (DR) [1]. The loss of PCs triggers the formation of microaneurysms as it causes the structural and metabolic weakening of the capillary wall [2]. Therefore, replacing these cells could represent an effective treatment in the future.

Adipose-derived stem cells (ASCs) are mesenchymal stem cells readily available from human or animal adipose tissue [3,4]. Their significant production of a plethora of growth factors, cytokines, antiapoptotic effects and their promotion of angiogenesis is now known [5,6]. In this regard, ASC transplantation has been shown to promote burn wound healing [7]. The promotion of retinal cell survival and regeneration, and the modulation of retinal inflammation and vascularization have been attributed to ASCs (reviewed in [8]). These protective properties improve the resistance of retinal cells to pathological insults, promoting their physiological recovery. In retinal vasculopathy that occurs in DR, ASCs improve vascularization by secreting pro-angiogenic factors that stimulate the formation of new blood vessels. They can differentiate into pericytes, stabilizing retinal vessels in model systems of retinal vasculopathy, and this demonstrates that they could be considered for regenerative cell therapy. In fact, in preclinical mouse models of retinopathy vasculopathy, when injected intravitreally, ASCs stabilize retinal microvessels, improving their functionality and providing an effective protective microenvironment in the retina of diabetic mice [9].

In our previous works, a successful PC-like differentiation was achieved by growing ASCs in a culture medium specifically designed for PCs [10]. Indeed, the use of ASCs offers several advantages since they are easily available and are suitable for autologous transplantation. They are being increasingly explored to treat a variety of human pathologies, including eye diseases [11].

Since PCs negatively modulate endothelial growth [12], their loss is directly involved in the pathogenesis of proliferative DR. Our previous study demonstrated that a HG condition directly damaged PCs through the activation of the PLA2/COX-2/VEGF-A pathway, a system that seems to act as a type of feed-forward loop between cPLA_2_/COX-2/PG and VEGF-A [13].

The development of the vascular system requires platelet-derived growth factors (PDGF) [14]. The PDGF-BB/PDGFR-β signaling pathway is essential for the cellular constitution and maturation of the blood–retinal barrier (BRB) through the recruitment of pericytes newly forming capillaries [15]. It has been reported that PDGF-BB is the main isoform of PDGF, being primarily expressed by ECs, while PDGFRβ is commonly expressed on PCs [16].

Under hyperglycemic conditions, there is a chronic imbalance in PDGFR-β-activated signaling pathways resulting in the loss of normal PC functions. PDGF-BB could, therefore, be a therapeutic target to maintain the physiological structure of retinal microcapillaries that are compromised in DR [17].

Phospholipases A_2_ (PLA_2_s) belong to a superfamily of enzymes hydrolyzing the *sn*-2 ester linkage in phospholipids, resulting in the release of lysophospholipids and free fatty acids [18,19]. The PLA_2_ isoforms are classified into three categories: cytosolic PLA_2_ (cPLA_2_) and secretory PLA_2_ (sPLA_2_), which require micromolar and millimolar levels of Ca^2+^, respectively, to express their activities, and Ca^2+^-independent PLA_2_ (iPLA_2_) [20]. The arachidonic acid (AA) resulting from the hydrolytic activity of PLA_2_s on membrane phospholipids is converted into prostaglandins (PGs), which play a variety of biological roles, including the modulation of inflammation. The conversion of AA to PGs occurs through the activity of cyclooxygenase (COX), followed by isomerization to PGs, such as PGE2, by prostaglandin E synthase [21]. Following inflammatory stimuli, cPLA_2_ catalyzes the release of AA through its Ca^2+^-dependent translocation to nuclear membranes and its activity is enhanced by phosphorylation of Ser-505 by ERK1/2 [22,23,24].

cPLA_2_ is well represented in adipose tissue and plays a crucial role in adipogenesis, in which its levels decrease, and the absence of phospholipase impairs the cell cycle progression occurring during adipogenesis. Moreover, cPLA_2_-deficient animals showed resistance to obesity when fed a high-fat diet [25].

In the aqueous humor of patients affected by DR, elevated concentrations of inflammatory cytokines, interleukin (IL)-1β and tumor necrosis factor alpha (TNF-α) have been found and their high levels were associated with the severity and prognosis of DR [26]. It has also been demonstrated that the anti-inflammatory, cytokine IL-10, is particularly elevated in the vitreous of patients with DR [27]. These fluctuating cytokine concentrations in patients with DR demonstrated that an imbalance between pro- and anti-inflammatory cytokines occurs and that their control could have beneficial effects on DR prognosis. Moreover, metalloproteases (MMPs) degrade basement membrane components of blood vessels, promoting angiogenesis. MMP-9 concentration has been shown to increase in the plasma of patients with proliferative DR [28].

Vascular endothelial growth factor (VEGF) is among the most important mediators involved in the pathogenesis and progression of DR [29], and its increased production/secretion induces proliferation and migration of microcapillary endothelial cells and a strong permeability increase in the blood–retinal barrier (BRB) [30]. The term, “VEGF” refers to a family of molecules comprising VEGF-A, VEGF-B, VEGF-C, VEGF-D, VEGF-E, VEGF-F and PGF [31]. Alternative splicing of the human VEGF-A gene results in six different isoforms, namely 121, 145, 165, 183, 189 and 206 [32], of which VEGF-A121 and VEGF-A165 are the most expressed in mammals. VEGF production and secretion are upregulated by various stimuli, including different isoforms of hypoxia-inducible factor 1 (HIF-1) [33] and insulin-like growth factor 1 (IGF-1), which is involved in retinal angiogenesis [34]. Physiologically, in the human retina, VEGF is produced by retinal pigment epithelial cells [35], astrocytes [36], Müller cells [37,38] and ECs [39]. The crucial role of vascular endothelial growth factor (VEGF) in the pathogenesis of DR has long been established, and anti-VEGF therapy via intravitreal injection is currently the mainstay in the management of DR [40].

Heme oxygenase (HO) catalyzes the heme degradation reaction, consuming oxygen to liberate ferrous iron, carbon monoxide (CO) and biliverdin IX [41]. Since it uses oxygen, HO could act in cases of oxidative stress by reducing it. Furthermore, CO and biliverdin have been shown to have antioxidant [42] and anti-inflammatory actions [43,44].

Since oxidative stress plays a central role in diabetic retinopathy [45], HO activity could represent an intracellular protective system to prevent damage induced by oxygen free radicals. The expression of the inducible HO-1 isoform is regulated by its substrate and cellular stressors, it is induced by a range of stimuli and its activity has been widely attributed a significant anti-inflammatory function [46], and numerous stimuli have been shown to have the capability of inducing HO-1 [47]. Moreover, it has been demonstrated that the overexpression of HO-1 plays a protective role in diabetic retinopathy due to its anti-apoptotic and anti-inflammatory effects in retinal ganglion cells [41].

In rats with streptozotocin-induced diabetes, it has been shown that HO-1 affected the level of glucose-evoked oxidative stress and played a role in the development of retinopathy through the Nrf2/ERK pathway [45]. In the ocular system, the silencing HO-1 gene expression in RPE suppressed the proliferation, migration and tube formation of co-cultured endothelial cells, indicating that HO-1 might have an angiogenic effect through the modulation of VEGF expression [48].

Inflammation is generally a response to disturbances in tissue homeostasis due to a variety of stimuli such as pathogens, tissue injury or contaminants resulting in the activation of innate and adaptive immunity. Inflammation involves a cascade of complex events [49]. In this context, accumulating evidence indicates that the transcription factor, Nrf2, plays a critical role in controlling the expression of antioxidant genes that ultimately exert anti-inflammatory functions [50]. The different domains present on Nrf2 regulate its activity under physiological and pathological conditions. In particular, the N-terminal domain binds it to its negative regulator Keap1, influencing its ubiquitination in the cytoplasm, while the Neh1 domain is responsible for its nuclear localization and regulates DNA binding [51]. Nrf2 is coupled to Keap1 and is destined for ubiquitination and destruction under normal homeostatic settings. Under stress, Nrf2 is released from Keap1 and moves to the nucleus where it attaches to antioxidant response elements (AREs), causing ARE-driven genes to begin to transcribe [52].

In vivo experiments demonstrated that Nrf2 plays a critical role in neointimal formation and vascular remodeling, having anti-inflammatory effects in the vascular system. Indeed, when ECs were exposed to TNF-α, the expression of Nrf2 increased, thus suppressing the transcriptional levels of monocyte chemotactic protein-1 and vascular cell adhesion molecule-1. These signaling pathways may help to repair and maintain the structural integrity of blood vessels, shedding light on Nrf2’s crucial functions in maintaining vascular homeostasis [53].

## 2. Results

### 2.1. ASC Pericyte-like Differentiation

**Figure 1:**
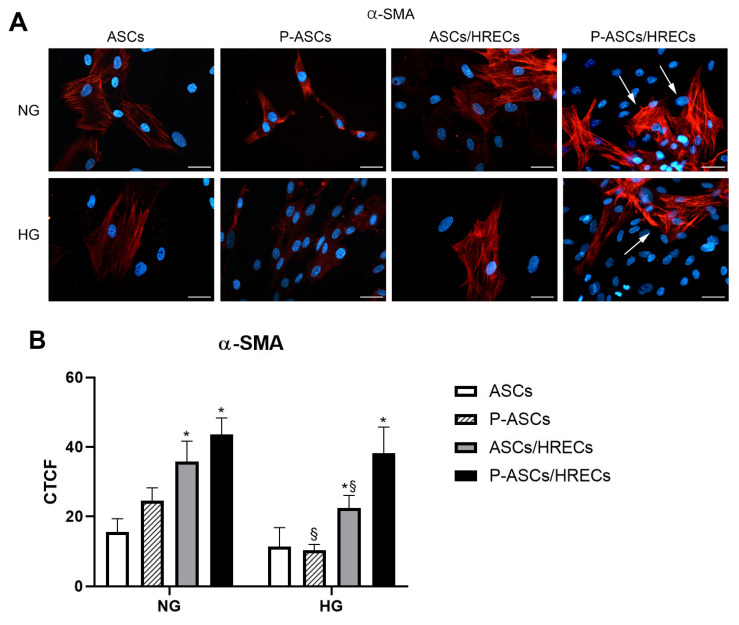
α-SMA immunostaining under different culture conditions of human ASCs and P-ASCs. (**A**) Microphotographs showing α-SMA expression levels (red) in ASCs or P-ASCs cultured alone (first two columns of photographs on the left) or in direct co-cultures with HRECs (last two columns of photographs on the right), or with HRECs, under NG or HG conditions. Blue fluorescence (DAPI counterstaining) indicates cell nuclei. Scale bar = 50 µm. Arrows: high levels of α-SMA expression. (**B**) Histograms showing fluorescence quantification data for α-SMA under the same conditions as above. Bars represent corrected total cell fluorescence (CTCF) mean values ± SD, obtained from at least three independent experiments. Statistically significant differences, determined by one-way ANOVA, followed by Dunnett’s multiple comparison test, are indicated as follows: * *p* < 0.05 vs. ASCs, at the same glucose concentrations; ^§^ *p* < 0.05 of each sample vs. the corresponding NG condition. Under NG conditions, the basal α-SMA expression in ASCs is increased when cultured in the pericyte medium. A further increase can be noticed when HRECs are also present, especially for P-ASCs, showing the typical filamentous pattern (arrows). A similar trend, although at slightly lower values, can be observed after glucose addition (HG). The lowest value is detectable in P-ASC monocultures.

Pericyte-like differentiation of human ASCs was evaluated by immunofluorescence expression of alpha-smooth muscle actin (α-SMA) and PDGFR-β, which are typical pericyte markers. Detections were carried out at day 4 of growth in ASC and P-ASC samples cultured under normal glucose (NG) or high glucose (HG) conditions, either alone or in the presence of HRECs. In ASC and P-ASC monocultures, basal α-SMA expression levels (Figure 1) were largely unchanged, whereas a marked increase was observed in the co-cultures with HRECs, both under NG and HG conditions, showing the typical filamentous pattern. Such an increase was more evident in P-ASC/HREC co-cultures, indicating their higher propensity to differentiate into PCs. These results confirm our previous data on ASC pericyte-like differentiation [54].

### 2.2. PDGFR-β and Phospho-PDGFR-β Immunofluorescence in ASCs and P-ASCs

**Figure 2:**
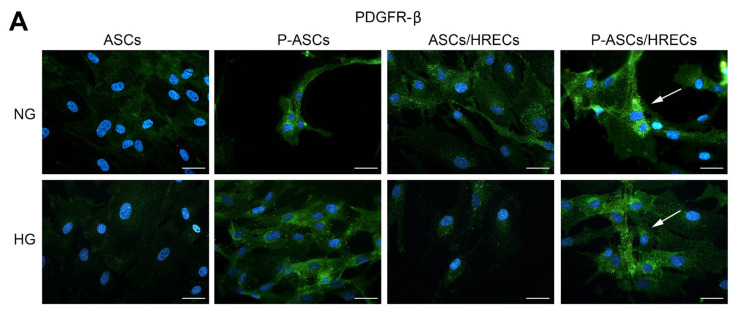

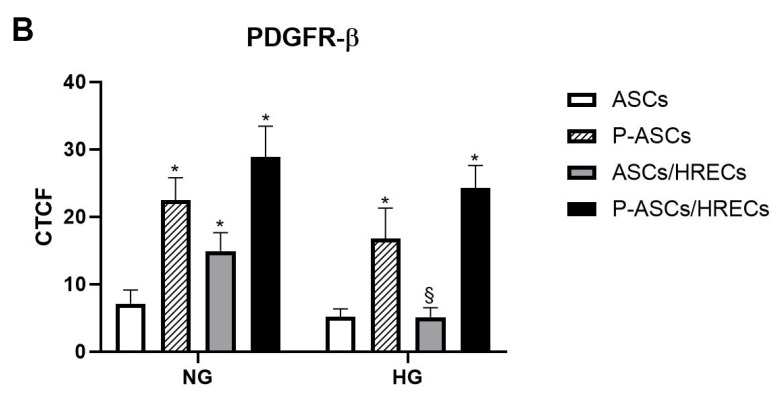
PDGFR-β immunostaining under different culture conditions of human ASCs and P-ASCs. (**A**) Microphotographs showing PDGFR-β expression levels (green) in ASCs or P-ASCs cultured alone (first two columns of photographs on the left) or in direct co-cultures with HRECs (last two columns of photographs on the right), or with HRECs, under NG or HG conditions. Blue fluorescence (DAPI counterstaining) indicated cell nuclei. Scale bar = 50 µm. Arrows: high levels of PDGFR-β expression. (**B**) Histograms showing fluorescence quantification data for PDGFR-β under the same conditions as above. Bars represent corrected total cell fluorescence (CTCF) mean values ± SD, obtained from at least three independent experiments. Statistically significant differences, determined by one-way ANOVA, followed by Dunnett’s multiple comparison test, are indicated as follows: * *p* < 0.05 vs. ASCs, at the same glucose concentrations; ^§^ *p* < 0.05 of each sample vs. the corresponding NG condition.

PDGFR is normally expressed on the pericyte surface. The interaction of this receptor with its ligand (PDGF-β) promotes the recruitment of pericytes to the vessel wall, ensuring both functional and structural BRB integrity. Therefore, by immunofluorescence assay, we evaluated the expression level of PDGFR-β (green emission) on ASCs and P-ASCs cultured alone or in the presence of HRECs either under normal or high glucose conditions. By analyzing PDGFR-β immunostaining (Figure 2), it could be noticed that a very low level of PDGFR-β immunoreactivity was detectable in ASC cultures, either under NG or HG conditions. These basal levels were significantly higher in P-ASCs, supporting a pericyte-like differentiation process. Under NG conditions, an increase was also observed when ASCs or P-ASCs were cultured with HRECs. Under HG conditions, PDGFR-β expression levels were consistently lower, and marked increases were only observed in P-ASCs/HREC co-cultures. It can be concluded that, even under hyperglycemic conditions, such as those occurring in a diabetic eye, the presence of P-ASCs could preserve the interaction of this receptor with its ligand. Under NG conditions, the basal PDGFR-β expression in ASCs was markedly increased when cultured in the pericyte medium. An increase can also be noticed when HRECs are present, especially for P-ASCs. Generally, lower values can be observed after glucose addition in each sample, especially in ASCs/HRECs.

### 2.3. PDGFR-β and Phospho-PDGFR-β Western Blot Analyses

During new vessel formation, the PDGF-B/PDGFR-signaling pathway is crucial for the recruitment of pericytes [55]. In a recent investigation, we evaluated PDGF-B mRNA levels in HRECs, in NG and in HG, grown in monoculture or in co-culture with ASC or P-ASC. We demonstrated that ASCs and especially P-ASCs induced an increase in HREC production of PDGF-B mRNA levels, also under HG conditions [54].

**Figure 3:**
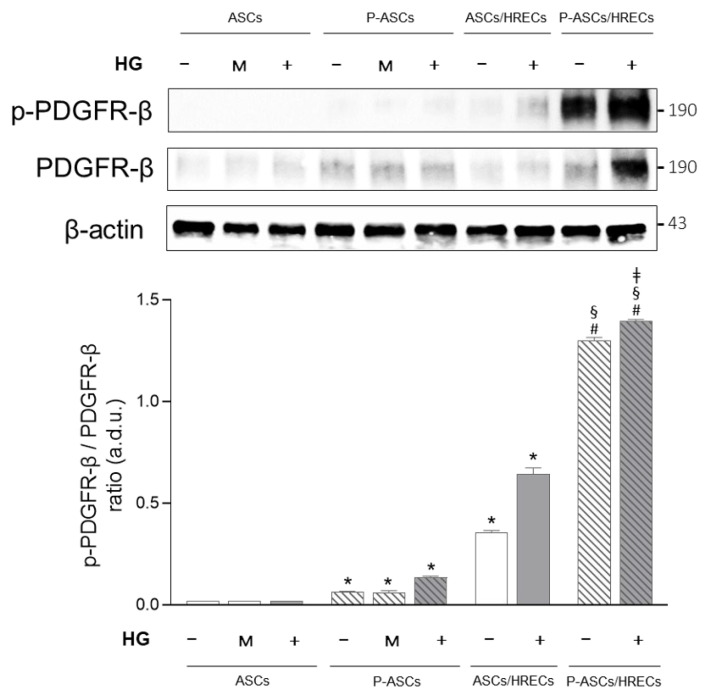
Evaluation of PDGFR-β/p-PDGFR-β in human adipose-derived mesenchymal stem cells (ASCs) and in human pericyte-like ASCs (P-ASCs) co-cultured with human retinal endothelial cells (HRECs) (indirect co-cultures). Data were gathered using Western blot analysis from samples that were cultured either under normal glucose (NG), mannitol (M) or high glucose (HG) acute conditions. Immunoblot analyses were performed on ASC and P-ASC lysates using specific antibodies against PDGFR-β and activated PDGFR-β (phosphorylated, p-PDGFR-β). β actin was used to verify the equal loading of 30 μg of protein per lane. Image J software (version 1.52a, NIH, Bethesda, MD, USA) was used to carry out densitometric analysis of the immunoblots, indicating the protein quantification of each band (in arbitrary densitometry units, a.d.u.). p-PDGFR-β/PDGFR-β ratios are reported in the graph. The bars represent means ± SD of three independent experiments performed in triplicate. Statistically significant differences determined by one-way ANOVA, followed by Dunnett’s multiple comparison test, are indicated as follows: * *p* < 0.05 vs. ASC monocultures in NG and HG; # *p* < 0.05 vs. P-ASC monocultures in NG and HG; ^§^ *p* < 0.05 vs. ASC/HREC co-cultures in NG and HG; ^‡^ *p* < 0.05 vs. P-ASCs/HRECs co-cultures in NG.

Here we evaluated the expression of the PDGF-β receptor (PDGFR-β) and activated phospho-PDGF-β receptor (p-PDGFR-β) in ASCs and P-ASCs in co-cultures with HRECs (Figure 3). ASC monocultures expressed total PDGFR-β without any difference among NG, mannitol and HG treatments. In P-ASC monocultures, total PDGFR-β expression was higher than for ASCs, without any significant difference among the treatments. In ASCs co-cultured with HRECs, total PDGFR-β expression was unchanged in comparison to the respective monocultures. In P-ASCs co-cultured with HRECs, a slight increase in total PDGFR-β expression in comparison to the respective monocultures was found. Under acute HG conditions, the expression of the total PDGFR-β expression was enhanced in comparison to NG conditions. The active form of PDGFR-β (p-PDGFR-β) was found in in P-ASC monocultures and in co-cultures of ASCs/HRECs and P-ASCs/HRECs. The p-PDGFR-β/PDGFR-β ratio was 2.23-fold higher in P-ASC monocultures in respect to the same cells exposed to NG or mannitol.

Moreover, HRECs induced in co-cultured ASCs showed a significant increase in the p-PDGFR-β/PDGFR-β ratio in NG and, even more, in HG.

In P-ASCs co-cultured with HRECs, the activated/total PDGFR-β ratio increased significantly by approximately 21-fold in NG and by approximately 10-fold in HG compared to P-ASCs in monocultures under the same experimental conditions. In P-ASCs/HRECs in the presence of acute HG conditions, the p-PDGFR-β/PDGFR-β ratio increased by almost 1.1-fold in comparison to the same co-cultures in NG medium.

The high expression of activated p-PDGFR-β, particularly in P-ASCs, confirms their crosstalk with HRECs, thus guaranteeing the recruitment of these cells when, under HG conditions, PCs detach from the retinal microcapillaries. The presence of these PC-differentiated cells, which would replace PCs, could maintain the stability of the vessel, thus avoiding that the loss of control over the endothelium by the PCs, an event that occurs in diabetes, could lead to endothelial proliferation and vessel permeability.

### 2.4. Cytoplasmic and Nuclear Nrf2 Protein Expression

**Figure 4:**
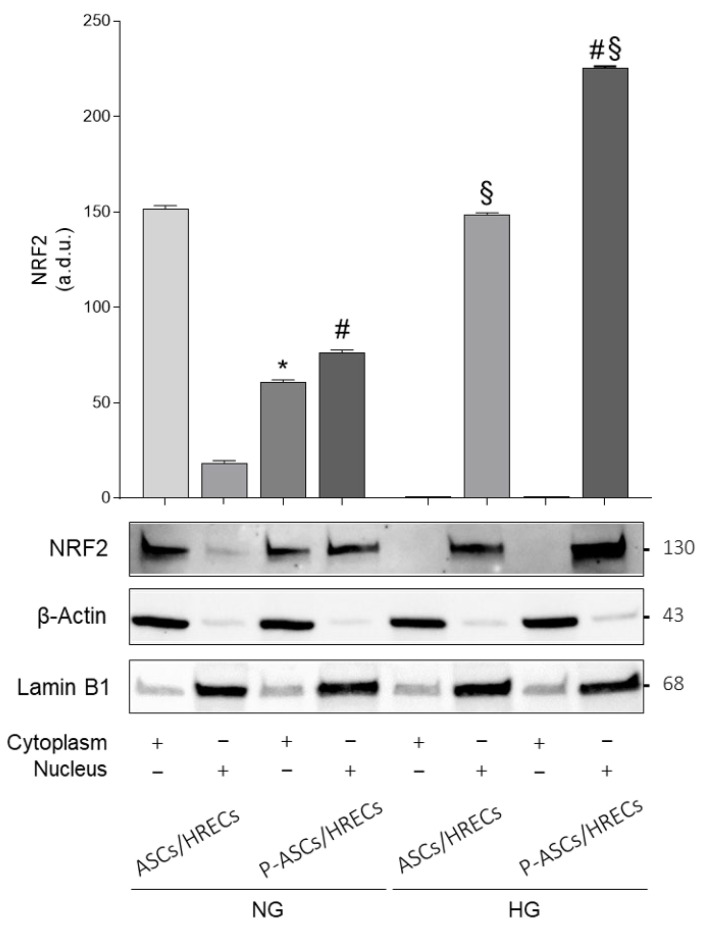
Evaluation of Nrf2 in the cytoplasm and nuclei of human adipose-derived mesenchymal stem cells (ASCs) and in human pericyte-like ASCs (P-ASCs) co-cultured with human retinal endothelial cells (HRECs) (indirect co-cultures). Data were gathered using Western blot analysis from samples that were cultured either under normal glucose (NG) or acute high glucose (HG) conditions. β-Actin and Lamin B1 were used to verify the equal loading of 30 μg of protein per lane in cytoplasm and nuclei, respectively. Image J software was used to carry out densitometric analysis of the immunoblots, indicating protein quantification of each band (in arbitrary densitometry units, a.d.u.). Quantitative analysis of Nrf2 was normalized to β-Actin and Lamin. The bars represent means ± SD of three independent experiments performed in triplicate. Statistically significant differences, determined by one-way ANOVA, followed by Dunnett’s multiple comparisons test, are indicated as follows: * *p* < 0.05 vs. cytoplasmic fraction of ASCs/HRECs in NG; # *p* < 0.05 vs. nuclear fraction of ASCs/HRECs in NG or HG; ^§^ *p* < 0.05 vs. corresponding nuclear fractions in NG.

In Figure 4, the evaluation of Nrf2 in cytoplasm and nuclei of human ASCs and in human P-ASCs co-cultured with HRECs is shown. In ASCs, grown in NG, the protein levels of the cytoplasmic fraction are 2.5-fold higher than in P-ASCs, and the protein levels of the nuclear fraction in P-ASCs are 4.1- and 1.5-fold higher than in ASCs, in NG and HG, respectively. Moreover, co-cultures of P-ASC with HREC showed a higher expression of nuclear Nrf2 protein levels, 8.0- and 7.0-fold higher than the corresponding nuclear fraction in NG. Mannitol (osmotic control) did not induce any change in Nrf2 translocation. Mannitol (osmotic control) did not induce any change in Nrf2 translocation (Supplementary Figure S1).

Upregulation and nuclear translocation of Nrf2 under acute HG conditions in ASC and P-ASC co-cultures may suggest their protective role against oxidative stress via the regulation of antioxidant gene expression. Acute HG conditions induced the activation of Nrf2 that enters the nucleus and binds to the antioxidant responsive element (ARE), thus initiating the transcription of gene encoding for antioxidant enzymes and contributing to the anti-inflammatory process [56].

### 2.5. HO-1 Immunofluorescence

**Figure 5:**
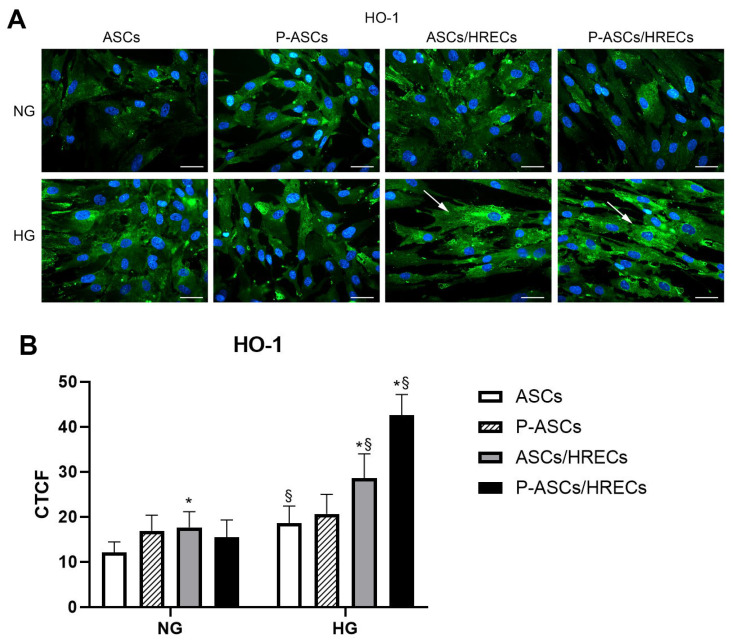
Heme Oxygenase-1 (HO-1) immunocytochemical expression in human ASC and P-ASC cultures under NG and HG conditions. (**A**) Microphotographs showing immunocytochemical HO-1 expression levels (green) in ASCs or P-ASCs cultured alone (first two columns of photographs on the left) or in direct co-cultures with HRECs (last two columns of photographs on the right), or with HRECs (ASCs/HRECs, P-ASCs/HRECs) under NG or acute HG conditions. Blue fluorescence (DAPI counterstaining) indicates cell nuclei. Scale bar = 50 µm. Arrows: high levels of HO-1 expression. (**B**) Histograms show the cell fluorescence quantification for HO-1 immunostaining under the same conditions as indicated above. Bars represent the corrected total cell fluorescence (CTCF) mean values ± SD, obtained from three independent experiments performed in triplicate. Statistically significant differences, determined by one-way ANOVA, followed by Dunnett’s multiple comparison test, are indicated as follows: * *p* < 0.05 vs. ASCs, at the same glucose concentrations; ^§^ *p* < 0.05 of each sample vs. the corresponding NG conditions. Overall, increased HO-1 expression can be observed under high glucose (HG) conditions, particularly when ASCs or P-ASCs are co-cultured with HRECs (arrows).

In Figure 5, the immunocytochemical expression of HO-1 in human ASC and P-ASC monocultures and in co-culture with HRECs under NG and HG conditions is shown. HO-1 basal expression was detected in mono- and co-cultures under NG conditions. The cell fluorescence quantification highlights an increase in HO-1 in ASCs and P-ASCs in co-cultures with HREC in the presence of HG by about 1.6-fold and 2.8-fold, respectively, compared with the same cells in NG, with a greater increase in P-ASCs compared with ASCs (1.4-fold).

### 2.6. cPLA_2_ Specific Activity and HO-1 Protein Content in ASCs and P-ASCs

In Table 1, cPLA_2_ specific activity values and HO-1 protein content in ASCs and P-ASCs are shown. Under NG conditions, cPLA_2_ activity values were similar for ASC and P-ASC, both in mono- and co-cultures with HREC. When maintained in HG medium, cPLA_2_ specific activities increased by 3.5-fold in ASCs and by 2.15-fold in P-ASCs, highlighting a significant inflammatory response of both cell types to the hyperglycemic insult; however, it was much less pronounced in P-ASC. The presence of co-cultured HRECs under acute HG conditions significantly reduced the phospholipase activity in ASCs by almost 29%, while, interestingly, it was reduced almost to control values (NG) in P-ASCs. These data highlighted the inflammatory response of P-ASCs to the hyperglycemic insult, significantly lower than that of ASCs. This sort of “increased resistance” to acute HG exposure confirmed our previous studies [57]. Furthermore, the contact with HRECs in Transwell systems partially protected ASCs, but almost completely protected P-ASCs, from HG-evoked/cPLA_2_-mediated inflammatory damage.

In ASCs and P-ASCs under NG conditions, in mono- and co-cultures with HREC, the HO-1 protein content remained unchanged. When exposed to HG, intracellular HO-1 significantly increased in both ASCs and P-ASCs in monoculture when compared with respective control cells in NG (by 1.36- and 1.19-fold, respectively). Interestingly, co-culture conditions in HG caused significant HO-1 increases by 1.76-fold in ASCs and 3.26-fold in P-ASCs compared with the respective monocultures, maintained in HG.

Treatments with the same concentrations of mannitol (osmotic control) did not induce any change in enzymatic activity and HO-1 content.

These data confirmed, again, the capacity of ASCs and differentiated P-ASCs to oppose hyperglycemic stress conditions by activating antioxidant molecular systems [57] such as HO-1 activity. The increased intracellular concentration of HO-1 in ASCs and, even more so, in P-ASCs, both co-cultured with HRECs, showed the significant induction of enzyme activity by the latter, indicating an effective crosstalk between the two cell types under opposing hyperglycemic stress, mostly reinforced by HRECs on P-ASCs.

### 2.7. VEGF-A and PGE2 Levels in Media from ASC and P-ASC Monocultures and Co-Cultures with HRECs

Table 2 shows the VEGF-A (henceforth referred to as VEGF) and PGE2 levels from mono- and co-culture media. Supernatants from HREC monocultures were taken into account, both in NG and HG, to quantify the contribution of the endothelium in the co-culture systems.

Confirming previous data [58], in NG medium, differentiated ASCs produced 34% less VEGF than their cellular progenitors. In NG, the VEGF levels in the ASC/HREC co-cultures could be attributable to the sum of the amounts of growth factor produced by both the ASCs and HRECs in monoculture, while the amount of VEGF was 23% lower in the supernatants from the P-ASC/HREC co-cultures. As expected, and confirming previous data [39,59], when exposed to acute HG concentrations, HRECs doubled their secretion of VEGF in the medium and, similarly, the growth factor produced by ASCs was approximately 1.6-fold higher than by NG-ASCs. On the other hand, in P-ASC monocultures, the increase in VEGF production was slightly reduced (by 1.4-fold) compared to ASC monocultures. In HG ASCs/HRECs, the levels of VEGF showed a significant increase with respect to NG (1.34-fold higher), due to the synergistic effect of the single cellular components stimulated by HG. The amount of VEGF released in the hyperglycemic medium by P-ASCs/HRECs was similar to the corresponding one in NG, reflecting the different behavior of ASCs and P-ASCs when exposed to HG concentrations.

PLA2 is the rate-limiting step in prostaglandin production by hydrolyzing arachidonic acid from membrane phospholipids; thus, PGE2 secretion in media was quantified. ASCs produced and secreted discrete levels of PGE2 under NG conditions (almost 1.4-fold less than HREC), confirming previous data according to which PGE2 contributes to the maintenance of the self-renewal capacity of mesenchymal stem cells from adipose tissue [60]. In NG, the amount of PGE2 detected in supernatants from both ASC/HREC and P-ASC/HREC co-cultures corresponded to the sum of contributions of single cells in monocultures. Confirming our previous data [39], in HRECs the secretion of PGE2 in acute HG increased by almost 1.6-fold more than in NG. In ASCs, the PGE2 secretion increased by almost 1.9-fold, demonstrating that the hyperglycemic stimulus is a pro-inflammatory one for ASCs as well, while in P-ASCs the prostaglandin level was similar to that which occurred in NG (1.1-fold with respect to NG). Supernatants from ASC/HREC co-cultures revealed a significant, almost 2-fold, increase in PGE2 concentration compared with the respective conditions in regard to NG, greater than the sum of PGE2 levels in HRECs and ASCs alone. The PGE2 amount in media from P-ASC/HREC co-cultures was, interestingly, similar to that found in the respective NG co-cultures; however, it was two-fold lower than the ASC/HREC co-cultures. These data confirmed the same trend of phospholipase A2 activation and, again, the different behavior of ASCs and P-ASCs in relation to HG concentrations, P-ASCs being less responsive, in terms of inflammatory response, to HG concentrations simulating, in vitro, the diabetic retinopathy environment. Treatments with the same concentrations of mannitol (osmotic control) did not induce any change in VEGF and PGE2 levels.

### 2.8. High Glucose Effects on mRNA Levels of Pro-Inflammatory Cytokines, Interleukin-10 and Angiogenic Factors in ASCs and P-ASCs Co-Cultured with HRECs

**Figure 6:**
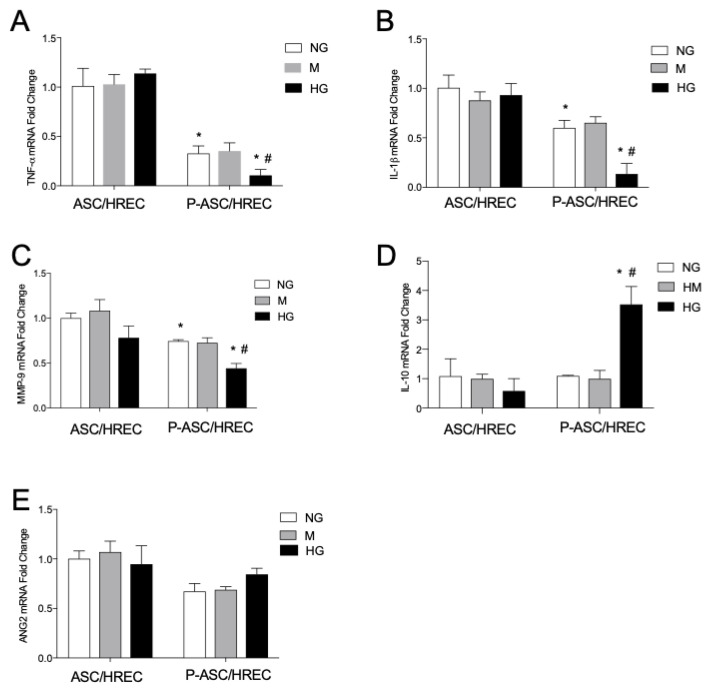
Evaluation of mRNA levels of cytokines related to inflammation in human adipose-derived mesenchymal stem cells (ASCs) and in human pericyte-like ASCs (P-ASCs) co-cultured with human retinal endothelial cells (HRECs) (indirect co-cultures). Data were gathered using qRT-PCR analysis from samples that were cultured either under normal glucose (NG), mannitol (M) or high glucose (HG) conditions. (**A**) tumor necrosis factor alpha (TNF-α) mRNA levels; (**B**) interleukin (IL)-1β mRNA levels; (**C**) IL-10 levels; (**D**) metalloproteases (MMP)-9 mRNA levels; (**E**) angiopoietin-2 (ANG-2) levels. The bars represent means ± SD of three independent experiments performed in triplicate. Statistically significant differences determined by one-way ANOVA, followed by Dunnett’s multiple comparisons test, are indicated as follows: * *p* < 0.05 vs. the same cultures in NG; # *p* < 0.05 vs. ASCs in HG.

The mRNA levels of cytokines related to inflammation were measured using quantitative RT-PCR analyses in HRECs co-cultured with ASCs and P-ASCs, both under NG and HG conditions (Figure 6).

Similar values of TNF-α mRNA levels were detected in ASCs under NG and HG conditions (panel A). In P-ASCs, the mRNA value was markedly lower under NG conditions with respect to ASCs (by 3.3-fold) and was even more significantly reduced by nearly 34% in HG with respect to normal conditions.

The same fold-change trend was observed for IL-1β mRNA levels (panel B): in fact, no evident differences were noticeable in ASCs, both under NG and HG conditions. In P-ASCs, IL-1β m-RNA was almost 40% lower in NG vs. ASCs, and, when exposed to HG, a significant 75% reduction was observed with respect to NG conditions.

No changes in mRNA expression for IL-10 were found in ASCs, except for a slight, but not significant, reduction in HG conditions (panel C). Conversely, significant dynamic effects were observed for the anti-inflammatory cytokine mRNA levels in P-ASCs under the different conditions. In particular, whereas the mRNA expression in NG medium was similar to ASCs under the same conditions, a strong 3.6-fold increase was detected in P-ASCs exposed to acute HG.

In further experiments, mRNA expression levels of angiogenic factors MMP9 and angiopoietin-2 (ANG-2) were evaluated. In co-cultured ASCs in NG, the MMP-9 mRNA levels were similar, with a not significant reduction under HG conditions (panel D). Again, a modulation in mRNA expression was found in P-ASCs, with an approximately 32% decrease in NG compared with the progenitor ASCs, and a significant almost 1.7-fold decrease in hyperglycemic medium. Mannitol did not change mRNA levels of pro-inflammatory cytokines, interleukin-10 and angiogenic factors.

The data reported indicate a significant modulation of inflammation-related genes by HRECs on P-ASCs, but not on ASCs, under acute hyperglycemic conditions in favor of an anti-inflammatory phenotype. Furthermore, a decrease in the expression of the angiogenic factor, MMP-9, was observed.

## 3. Discussion

In the last few decades, ASCs have been widely investigated for their multipotent differentiation ability. In fact, they can give rise not only to cells of mesodermal origin [61] but can also transdifferentiate towards cells of different line types such as neurons or glial cells [62].

It has already been demonstrated that when pericytes interact with endothelial cells, their secretion of growth factors, such as TGF-β or PDGF, induces a switch toward the contractile phenotype [63,64,65]. Moreover, it is well-known that mesenchymal cells express smooth muscle cell markers when co-cultured with endothelial cells, thus inducing precursor cells to differentiate into pericytes or smooth muscle cells [66]. It has also been reported that a significant increase in αSMA fibrous expression occurs in ASCs located in close proximity to human retinal endothelial cells [67], clearly indicating a crosstalk between the two cell types.

In our previous study, we demonstrated in HRECs that, under both NG and HG conditions in the P-ASC/HREC co-cultures, a significant simultaneous increase in PDGF-B mRNA expression and marked increases in PDGFR-β mRNA in ASCs and P-ASCs occurred [54].

In this study, our results demonstrate that PDGFR-β and its active form, p-PDGFR-β are expressed in ASCs and, even more, in P-ASCs co-cultured with HRECs and that their expression in the presence of HG is higher than in NG, highlighting that both cell types express one of the most significant markers of PCs and confirming their differentiation into pericyte-like cells. High expression of PDGFR and its phosphorylated active form, in particular in P-ASCs, under HG, highlighted by immunofluorescence assay and by Western blot analyses, indicated that these stem cells have the necessary characteristics to be recruited from HRECs to maintain a stable blood vessel, even under the pathological condition of DR, mimicked in our in vitro model.

In ASCs and in P-ASCs, co-cultured with HRECs, an elevated expression of Nrf2 was found more in the cytoplasmic fraction of ASCs and in the nuclear fraction of P-ASCs, under NG conditions and, even more, under acute HG conditions.

Therefore, HG conditions induced the activation of Nrf2, which enters the nucleus and, as known, binds to the antioxidant responsive element (ARE), thus initiating the transcription of gene encoding for anti-oxidant enzymes and contributing to the anti-inflammatory process [56].

It has been demonstrated that Nrf2 helps to control the heme oxygenase-1 (HO-1) axis, a strong anti-inflammatory target [68]. In a model of tight junction dysfunction, activation of the Nrf2/HO-1 axis does, in fact, result in a decrease in lipid peroxidation, TNF- and IL-6 levels, and an attenuation of the inflammatory response. Additionally, heme-degrading metabolites such as CO and bilirubin hasten the recovery from oxidative stress and inflammation [69].

If we consider that the BBB is not easily crossed by substances or drugs, and that P-ASCs, physiologically positioned in place of pericytes in the retinal microcapillaries, are capable of activating Nrf2 and the signal pathway which switches off inflammation, it can be understood how important it is to study the role played by these stem cells in DR, for their potential and future therapeutic applications.

Our results demonstrate that ASCs and P-ASCs have HO-1 in terms of protein content, which remained constant under NG conditions, both in mono- and co-cultures with HRECs. Both cell types, ASCs and P-ASCs, showed an interesting and remarkable increase in intracellular concentrations of HO-1 after HG treatment. This fact confirmed, once again, the “physiological tendency” to oppose hyperglycemic stress through the activation of antioxidant mechanisms of survival evoked by HO-1 activity (mostly highlighted in P-ASCs).

In an in vivo model of diabetic retinopathy, PLA_2_ was significantly activated, resulting in the breakdown of the blood–retinal barrier (BRB) caused by the damage to endothelial cells (ECs) and pericytes (PCs), both cellular components of retinal microvessels [70]. Our previous data from ECs/PCs co-cultures, treated with specific inhibitors of PLA_2_ isoforms, demonstrated that, in high or in fluctuating concentrations of glucose to mimic the diabetic condition, PLA_2_ activity, VEGF and PGE2 were significantly related to each other. Moreover, in a streptozotocin-induced diabetes rat model, the role of PLA_2_ in the regulation of VEGF and other important cytokines and adhesion molecules involved in BRB breakdown was shown [70]. Here, in NG, mono- and HREC-co-cultured ASCs and P-ASCs showed similar cPLA_2_ specific activity values. Otherwise, in HG medium, cPLA_2_ activity significantly increased in both cell-type monocultures, indicating the inflammatory response of both cells to the hyperglycemic condition (even if less in P-ASCs). When co-cultured with HRECs in HG, both ASCs and, even more P-ASCs, showed a reduction in cPLA_2_ activity. These data demonstrate a strong protective effect towards glucose inflammatory insult, exerted by ASCs and P-ASCs in co-cultures with HRECs.

The pathogenesis of DR is characterized by the impairment of the neurovascular unit, breakdown of the blood–retinal barrier (BRB), inflammation, perfusion deficiency, microvessel ischemia, and aberrant neoangiogenesis [71], events that trigger the production of pro-angiogenic molecules that mediate inflammation. Among these, VEGF-A plays a prominent role, inducing the modification of proteins involved in the formation of tight junctions with the consequent increase in microvessel permeability and in transcytosis [72]. Because of the central role of VEGF-A with respect to other isoforms, researchers and clinicians often refer to this subtype simply as VEGF [73].

We previously showed that VEGF-A-mediated glucose-induced damage in the retinal endothelium required the involvement of the ERK1/2/PLA2 pathway axis and, moreover, both the VEGF- and PLA_2_ inhibitors prevented the HG-induced damage, the impairment of tube formation on Matrigel and the expression of VEGF-A mRNA. Moreover, the VEGF inhibitor counteracted the HG-induced increase in activated ERK1/2 and cPLA_2_ [39].

In this study, under acute HG conditions, unlike the ASC/HREC co-cultures, the VEGF levels in P-ASC/HREC media were similar to the corresponding ones in NG. This different response to the hyperglycemic condition highlighted a different capacity of P-ASCs, compared with the progenitor ASCs, in the production of VEGF, probably even modulating the secretion of the growth factor by HRECs.

It has been demonstrated that the vitreous bodies from patients with proliferative diabetic retinopathy are characterized by high levels of PGE2, and significantly correlated with VEGF secretion, suggesting the potential therapeutic role for nonsteroidal anti-inflammatory drugs in retinal complications of diabetes [74]. Moreover, it has been shown that the PGE2/EP2 receptor pathway promotes the diabetic retinopathy in a rat model of diabetes [75].

In our results, in NG, ASCs secreted discrete PGE2 levels in media, less than HRECs under the same conditions, and this is in agreement with data reporting a regenerative bioactive role of these molecules in mesenchymal cells from adipose tissue [60]. The incubation in HG media elicited a significant inflammatory response both in ASC and HREC single cultures, evidenced by increased PGE2 release in supernatants, unlike P-ASCs, whose PGE2 value was almost similar to NG-incubation medium. Moreover, prostaglandin values in HG-ASC/HREC co-culture medium were doubled with respect to NG-co-cultured ones, unlike the values found in the HG-P-ASCs/HRECs media, which remained constant with respect to those quantified in the same co-cultures in NG. These findings confirm that, even in the presence of high concentrations of glucose, P-ASCs show a protective effect under acute hyperglycemic conditions [57].

It has been demonstrated that, during inflammation, a correlation between the Nrf2 pathway and prostaglandins may occur, providing evidence that PGE2 could act as a physiological regulator of Nrf2 or vice versa [76].

Our results, correlating Nrf2/COX/PGE2, demonstrated that in P-ASCs co-cultured with HRECs, the signal pathway, characterized by the migration of Nrf2 into the nucleus, is activated. In our opinion, this activation could lead to the final effect of the reduction in the release of inflammatory PGE2. This effect could play an important role in maintaining vessel integrity as it could block the inflammatory process induced by HG, which is the main cause of angiogenesis in diabetic retinopathy.

It has been demonstrated that the levels of proinflammatory cytokines are increased in serum or vitreous of DR patients [27]. It has also been reported that IL-10 is an anti-inflammatory cytokine that prevents angiogenesis by downregulating VEGF expression [77], so that its levels are lower in patients affected by proliferative DR than control subjects [78] and that the presence of high levels of IL-10 reduce the risk of DR in patients with DM [79,80].

In a previous study, we measured the mRNA levels of pro- and anti-inflammatory cytokines in ASC and P-ASC monocultures under NG and HG conditions and we demonstrated an increase in mRNA expression levels of the anti-inflammatory cytokine, IL-10, associated with a reduction in mRNA expression of pro-inflammatory cytokines, IL-1β and TNF-α [57]. Here, we confirmed those findings by measuring the mRNA levels of pro- and anti-inflammatory cytokines in ASCs and in P-ASCs co-cultured with HRECs. Our results suggest that differentiated P-ASCs are able to modulate the release of cytokines when in co-culture with HRECs, by reducing the synthesis of pro-inflammatory TNF-α and IL-1β and by increasing that of anti-inflammatory IL-10. Thus, antagonization of these inflammation-related cytokines by P-ASCs may have the role of protecting retinal microcapillaries in DR, highlighting the possibility of blocking their release by acting with a new therapeutic intervention in the treatment of DR.

Moreover, we demonstrated that in P-ASCs, in co-culture with HRECs, a modulation in the expression of MMP-9 and Ang2 mRNA was found, with a decrease in their release, compared with that by ASCs co-cultured with HRECs under hyperglycemic conditions.

Various studies have demonstrated the involvement of MMPs in the pathogenesis of DR [81]. A recent report demonstrated high levels of MMPs in the vitreous of diabetic patients, confirming that the risk of DR increases with high levels of MMP-9 [82].

The modulation of over-activated MMPs by differentiated P-ASCs could alleviate the damage to the retinal microcapillaries and could address an intriguing objective to hinder the events of DR.

In conclusion, numerous studies have been conducted on the possibility of cellular treatments as potential therapies for retinal diseases. However, there are still no data demonstrating their long-term efficacy and safety.

The main novelty, presented for the first time in this study, is the demonstration that P-ASCs are less responsive, in terms of inflammatory response, to HG concentrations. P-ASCs, in fact, show great resistance to hyperglycemic insult, in a model that simulates the in vitro conditions occurring in DR.

The results of this study give new evidence about the beneficial effects that P-ASCs exert on endothelial cells exposed to HG and confirm their potential therapeutic role in substituting retinal lost pericytes and restoring BRB integrity. Future studies may focus on describing the P-ASC mechanism of action in vivo.

## 4. Materials and Methods

### 4.1. Human Adipose Stem Cells

Human Adipose Mesenchymal Stem Cells (ASCs) were obtained from adipose tissue in the lipoaspirate harvested from the subcutaneous abdominal region of 3 healthy non-smoking female donors (28–32 years old). Liposuction procedures were carried out at Cannizzaro Hospital (Catania, Italy). All donors signed an informed consent form for using lipoaspirate for experimental procedures, which were carried out in accordance with the Declaration of Helsinki, after the protocol was approved by the local ethics committee (Catania Ethics Committee1; Authorization n. 398/202l/EMPO).

The lipoaspirate was washed with sterile phosphate buffer saline (PBS; Invitrogen, Monza, Italy) and incubated in Dulbecco’s Modified Eagle Serum-free Medium (DMEM; Sigma-Aldrich, Milan, Italy) containing 0.075% collagenase type I (Invitrogen, Monza, Italy), for three hours at 37 °C. An equal volume of DMEM containing 10% heat-inactivated fetal bovine serum (FBS; Gibco, Monza, Italy) was then added to inactivate the collagenase activity. After the digested lipoaspirate was centrifuged at 1200 rpm for 10 min, the pellet was resuspended in PBS and filtered through a 100 µm nylon cell filter (Falcon BD Biosciences, Milan, Italy). The cells were plated in T75 culture flasks (BD; Biosciences Falcon) with DMEM containing 10% FBS, 1% MSC growth supplement and 1% P/S. All cultures were expanded for 2–3 passages and plated for subsequent procedures. Their MSC nature was evaluated in some cell samples as previously reported [57]. Briefly, by immunocytochemistry and flow cytometry, their positivity for typical MSC markers (CD44, CD73, CD90 and CD105) and their immunonegativity for typical hematopoietic markers (CD14, CD34 and CD45) were verified.

A group of ASC samples was destined to pericyte-like differentiation according to a protocol previously adopted [54]. As previously reported, no significant difference was noticed between samples from different donors [83]. Pericyte-like ASCs (P-ASCs) were obtained by growing ASCs for 3 days in a culture medium specifically designed for pericytes (PM; Innoprot, Elexalde, Derio, Spain), containing 2% FBS. Following this treatment, immuno-expression of α-SMA and PDGFR-β (generally recognized as pericyte markers) was significantly increased compared with control ASC samples, and maintained in their basal medium (DMEM containing 2% FBS under NG conditions). Samples from each of the two culture groups (ASCs and P-ASCs) were then used for co-cultures with HRECs. ASCs or P-ASCs were used within passages 5–6.

### 4.2. Human Retinal Endothelial Cells

Human Retinal Endothelial Cells (HRECs) were purchased from Innoprot (P10880). HRECs were cultured in endothelial cell medium (ECM), with the addition of 5% FBS, 1% penicillin/streptomycin solution (P/S; ScienCell Research Laboratories, Milan, Italy) and 1% endothelial cell growth (ECGS). When about 70% confluency was reached, FBS was lowered to 2.5% for 24 h before further treatments: some cell samples were kept under NG conditions (5 mM) or 5 mM glucose plus 20 mM D-mannitol (osmotic control) whereas other samples were kept under HG conditions (25 mM), to evaluate the acute effect of high glucose. Both samples were cultured for an additional 48 h before being used for co-culture experiments.

### 4.3. Cell Co-Cultures

Two different co-culture experiments were performed to create an in vitro model of the BRB and to reflect physiological crosstalk between HRECs and P-ASCs/ASCs under NG and HG conditions.

Indirect co-cultures were arranged using multiwell plates with Transwell inserts, to mimic a BRB model system in vitro (Falcon permeable clear PET membrane inserts for six-well plates, 0.4 µm pore size; Falcon BD Biosciences).

First, HRECs were plated on the underside of the inserts at a density of 1.5 × 10^5^ cells/well and, after 4 h, the inserts were overturned inside the well. The following day, 25 mM glucose was added to half of the wells whereas the remaining ones were maintained in NG medium. After a further 2 days, ASCs or P-ASCs (7.5 × 10^4^ cells/well) were added on the top of the Transwell inserts, thus obtaining four indirect culture groups: ASCs/HRECs and P-ASCs/HRECs under NG conditions; ASCs/HRECs and P-ASCs/HRECs under HG conditions. Osmotic controls were also performed by incubating the co-cultures with 5 mM glucose plus 20 mM D-mannitol (M).

In this way, cells were in contact with each other through the pores of the filter but “physically” separated. This system allowed the preparation of separated ASCs and P-ASCs lysates, without any contamination from HRECs.

ASCs/HRECs were cultured in a mixed medium consisting of 50% ECM and 50% DMEM, whereas P-ASCs/HRECs were cultured in a medium consisting of 50% ECM and 50% PM. Monocultures of ASCs and P-ASCs were incubated under NG (5 mM), 5 mM glucose plus 20 mM D-mannitol (osmotic control) or HG (25 mM) conditions for the same times as co-cultures.

After four days of co-culture (acute hyperglycemic conditions), immunoblot analysis and qRT-PCR were performed on ASCs and P-ASCs, and compared with their respective monocultures, which were used as controls. Culture media were stored for subsequent ELISA analysis.

For immunostaining, direct co-cultures (mixed) of ASCs/HRECs or P-ASCs/HRECs were seeded on coverslips in multiwell plates and were treated with NG or HG for the same times above described. In these samples, HRECs were first plated onto coverslips allocated to multiwell plates (2 × 10^4^ cells/coverslip). The following day, 25 mM glucose was added to some samples to mimic hyperglycemic conditions, whereas other HREC samples were kept under NG conditions. After a further 48 h, ASCs or P-ASCs (5 × 10^3^ cells) were added to each HREC sample. Therefore, four co-culture groups were obtained: HRECs/ASCs and HRECs/P-ASCs under NG conditions; HRECs/ASCs and HREC/P-ASC co-cultures under HG conditions. HRECs/ASCs were cultured in a mixed medium consisting of 50% ECM and 50% DMEM, whereas HRECs/P-ASCs were cultured in a medium consisting of 50% ECM and 50% PM. The results were compared with their respective monocultures in NG or under HG conditions. After 4 days of co-culture, immunostaining for α-SMA and PDGFR-β was carried out to assess pericyte-like differentiation.

### 4.4. Fluorescence Immunocytochemistry Procedures

The immunocytochemical procedures used largely overlap those previously reported [57]. Briefly, cell cultures were fixed by 4% paraformaldehyde, washed and incubated for 30 min with a 5% solution of normal goat serum (Sigma-Aldrich) in PBS containing 0.1% Triton (Sigma-Aldrich). They were then exposed overnight to primary antibodies: mouse anti α-SMA (1:200; M0851, Dako, Milan, Italy) and rabbit anti PDGFR (1:100; ab32570, Abcam, Boston, MA, USA). The following day, cells were incubated for 60 min at room temperature with secondary antibodies conjugated with different fluorochromes: Cy3-conjugated goat anti-mouse (1:500; ab97035, Abcam) and FITC-conjugated goat anti-rabbit (1:500; ab6717, Abcam, Boston, MA, USA). The specificity of immunostaining was verified in control experiments by omitting the primary antibody. Finally, DAPI counterstaining was used to visualize cell nuclei (10 min).

Immunostaining quantification was carried out through the FIJI-Image J measuring tool (NIH, Bethesda, MD, USA). From each sample, at least three digital photomicrographs were randomly selected. From each photomicrograph, up to seven immunofluorescent cells were analyzed. Values were derived from the average grayscale intensity. The integrated density, the cell area and the mean fluorescence of the selected cells were estimated. Three replicated measurements were performed for each capture region. The same procedure was applied to three different background areas around the selected cell. Then, the Corrected Total Cell Fluorescence (CTCF) was calculated using the following equation: CTCF = integrated density − (cell area × background mean fluorescence).

### 4.5. Extraction of Total mRNA and Quantitative Real-Time Reverse Transcriptase Polymerase Chain Reaction (qRT-PCR)

After 4 days of co-culture, qRT-PCR was performed in ASCs and P-ASCs to determine the mRNA levels of IL-10, IL-1β, TNF-α, MMP9, ANG2 and 18S rRNA. Total cellular mRNA was extracted using QIAzol reagent (79306, QIAGEN Inc., Valencia, CA, USA) following the manufacturer’s instructions. By measuring the optical density at 260 and 280 nm, the concentration and purity of the RNA was evaluated using the nanodrop method. Transcription of first-strand mRNA (1 µg) in cDNA was performed using the QuantiTect Reverse Transcription Kit (205313, QIAGEN Inc.).

The cDNAs were amplified in parallel reactions using iTaq Universal SYBR Green Supermix (1725124, Bio-Rad, Milan, Italy) with 0.8 µM primers provided by Eurofins Genomics Germany GmbH (Ebersberg, Germany) in one final volume of 10 µL. Amplifications were performed in a 7300 real-time PCR system (Applied Biosystems, Thermo Fisher Scientific, Waltham, MA, USA). The qRT-PCR data were analyzed in triplicate and normalized by the expression of the endogenous 18S rRNA gene using the comparative threshold cycle method (ΔΔCt).

The specific set of primers is reported in Table 3.

### 4.6. Immunoblot Analyses

After treatment of Transwell co-culture with NG, M and HG, cells were harvested with a cell rake after being washed twice with PBS. Subsequently, they were centrifuged (1000 rpm × 5 min. at 25 °C). After elimination of the supernatant, the pellets were lysed with RIPA buffer (20188, EMD Millipore Corporation, Temecula, CA, USA) containing cocktails of protease inhibitors (Protease Inhibitor Cocktail Set III EDTA—Free, 539134, EMD Millipore Corporation) and phosphatase (Phosphatase Inhibitor Cocktail 2, P5726, and Phosphatase Inhibitor Cocktail 3, P0044, Sigma-Aldrich, St. Louis, MO, USA). Subsequently, they were sonicated and centrifuged (13,000 rpm × 20 min. at 4 °C). Protein concentrations were calculated by the BCA protein assay (BCA Protein Assay Kit; sc-202389, Santa Cruz Biotechnology, Santa Cruz, CA, USA).

We then separated 20 μg of the protein extracts by electrophoresis on 4–20% prefabricated polyacrylamide gels (Mini-PROTEAN^®^ Prefabricated Protein Gels TGXTM; 4561096, Bio-Rad Laboratories, Segrate, Italy), and electrotransferred onto nitrocellulose membranes (Packs 0.2 μm Trans-Blot Turbo Mini Nitrocellulose Transfer Tube; 1704158, Bio-Rad Laboratories). The membranes were blocked for 1 h in blocking buffer (LI-COR Biosciences, Lincoln, NE, USA) and incubated at 4 °C overnight with the primary antibodies: Phospho-PDGFR-β (1:1000; ab62367, Abcam Boston, MA, USA), Total PDGFR-β (1:1000; ab69506, Abcam), Nrf2 (1:1000; ab137550, Abcam) and β-Actin (1:1000; ab8226, Abcam) and Lamin B1 (1:1000; 12586, Cell Signaling, Danvers, MA, USA) as loading controls. After performing three washings (5 min each), the membranes were incubated for 1 h at room temperature with the secondary antibody (Abcam) and detected by enhanced chemiluminescence (ECL Super-Signal West Dura Extended Duration Substrate; 34075, Thermo Fisher Scientific) using the Chemi-Doc touch imaging system (Bio-Rad, Hercules, CA, USA). Densitometric analyses of the blots were performed using Image J software (National Institutes of Health, Bethesda, MD, USA).

### 4.7. Subcellular Fractionation

Subcellular fractionation was carried out to obtain the cytoplasmic and nuclear fractions by using the NE-PER^®^ kit (Pierce, Rockford, IL, USA) following the manufacturer’s instructions. The concentrations of the lysates were obtained by means of the BCA calibration curve. Subsequently, 20 μg of proteins was loaded onto polyacrylamide gels, separated by electrophoresis and transferred onto a nitrocellulose membrane. The membranes were incubated overnight with Nrf2 and the following day, after appropriate washings, the secondary antibody was added for 1 h. For detection, the Chemi-Doc touch imaging system was used and densitometric analyses of the blots were performed using Image J software.

### 4.8. cPLA_2_ Activity and HO-1 Content

For PLA_2_ activities (cPLA_2_ assay kit, Cayman, 765021 Ann Arbor, MI, USA), equal amounts of cell lysates were incubated in a 96-well plate with the substrate arachidonoyl thio-phosphatidylcholine (ATPC), as previously reported [84]. The results were expressed as pmol of ATPC hydrolyzed per minute and per milligram protein (pmol/min/mg). Each sample was analyzed in triplicate.

For HO-1 content (Abcam ELISA Kit, ab207621), equal amounts of cell lysates were incubated in a 96-well plate with Human Heme Oxygenase 1 Capture Antibody. The results are expressed as ng/mL. Each sample was analyzed in triplicate.

### 4.9. VEGF and PGE2 Release

Supernatants of cell mono- and co-cultures were collected and aliquots were used for PGE2 and VEGF estimations, using commercially available kits, following the manufacturer’s instructions (PGE2 by kit from Cayman Chemicals Co., Ann Arbor, MI, USA; VEGF by kit from R&D Systems Inc., Minneapolis, MN, USA). For PGE2, the detection range was 7.8–1000 pg/mL, whereas for VEGF, it was 20–2500 pg/mL. Each sample was analyzed in triplicate.

### 4.10. Statistical Analysis

Experiments were performed three times (n = 3) in triplicate (biological and technical replicates). Data are reported as mean ± SD. The different groups were compared by one-way or two-way analysis of variance (ANOVA), as reported in the figure legends. A *p* value < 0.05 was considered a statistically significant difference between the experimental and control groups. Statistical analysis and graph design were carried out by means of GraphPad Prism 9 software (GraphPad Inc., San Diego, CA, USA).

## Figures and Tables

**Table 1:** cPLA_2_ specific activity and HO-1 content in lysates from ASCs and P-ASCs in mono- and co-cultures with HRECs (indirect co-cultures), under NG, M and HG conditions.

Mono-/Co-Cultures	cPLA_2_ Activity (pmol/min/mg)	HO-1 (ng/mL)
**Normal Glucose**		
ASCs	19.3 ± 1.01	0.72 ± 0.05
P-ASCs	18.4 ± 1.3	0.80 ± 0.06
ASCs/HRECs	20.1 ± 1.24	0.83 ± 0.04
P-ASCs/HRECs	22.3 ± 1.37	0.78 ± 0.05
**Mannitol**		
ASCs	17.8 ± 1.9	0.68 ± 0.07
P-ASCs	19.8 ± 1.6	0.84 ± 0.09
ASCs/HRECs	21.4 ± 2.11	0.75 ± 0.08
P-ASCs/HRECs	24.7 ± 2.25	0.86 ± 0.07
**High Glucose**		
ASCs	67.5 ± 4.03 *	0.91 ± 0.06 *
P-ASCs	39.7 ± 2.3 *^§^	0.95 ± 0.09 *
ASCs/HRECs	48.2 ± 3.16 *^‡^	1.6 ± 0.1 *^‡^
P-ASCs/HRECs	28.0 ± 2.23 *^‡†^	3.1 ± 0.4 *^‡†^

cPLA_2_ activity and HO-1 content were measured in whole cell lysates, following the manufacturers’ instructions. D-Mannitol (M) was used as an osmotic control. Values (means ± S.E.M.) are from three independent experiments (n = 3). Statistically significant differences, determined by one-way ANOVA, followed by Dunnett’s multiple comparison test, are indicated as follows: * *p* < 0.05 vs. respective cells under NG or M conditions; ^‡^ *p* < 0.05 vs. the same cell type in monoculture, in HG; ^§^ *p* < 0.05 P-ASCs vs. respective ASCs; ^†^ *p* < 0.05 P-ASCs/HRECs vs. respective ASCs/HRECs, in HG.

**Table 2:** VEGFA and PGE2 determinations in media from mono- and co-cultures (indirect co-cultures) under NG, M and HG conditions.

Mono/Co-Cultures	VEGFA (pg/mL)	PGE_2_ (pg/mL)
**Normal Glucose**		
HRECs	36 ± 1.8	84 ± 6.1
ASCs	91 ± 7.2	125 ± 9.8
P-ASCs	60 ± 4.2 ^§^	90 ± 5.3 ^§^
ASCs/HRECs	142 ± 12.0	214 ± 11.2
P-ASCs/HRECs	109 ± 10.1 ^†^	181 ± 8.9 ^†^
**Mannitol**		
HRECs	41 ± 2.9	91 ± 8.0
ASCs	87 ± 6.4	132 ± 10.2
P-ASCs	69 ± 5.1 ^§^	91 ± 7.6 ^§^
ASCs/HRECs	126 ± 10.7	222 ± 10.9
P-ASCs/HRECs	98 ± 8.4 ^†^	177 ± 9.5 ^†^
**High Glucose**		
HRECs	71 ± 5.1 *	133 ± 9.1 *
ASCs	142 ± 8.4 *	234 ± 10.8 *
P-ASCs	85 ± 5.3 *^§^	104 ± 7.9 ^§^
ASCs/HRECs	191 ± 16.4 *	417 ± 22.8 *
P-ASCs/HRECs	112 ± 10.8 ^†^	216 ± 16.6 *^†^

Cell culture media from mono- and co-cultures were assayed for VEGFA and PGE2 release, following the manufacturers’ instructions. D-Mannitol (M) was used as an osmotic control. Values (means ± S.E.M.) are from three independent experiments (n = 3). ANOVA and Dunnett’s multiple comparison test were used to compare VEGF and PGE2 values. * *p* < 0.05 vs. respective cells under NG or M conditions; ^§^ *p* < 0.05 P-ASC vs. respective ASC monoculture media, in NG, M or HG; ^†^ *p* < 0.05 P-ASCs/HRECs vs. respective ASCs/HRECs co-culture media, in NG, M or HG.

**Table 3:** Set of primers used for qRT-PCR.

Gene	Sequence (5′-3′)
IL10	Fw: GACTTTAAGGGTTACCTGGGTTG
	Rv: TCACATGCGCCTTGATGTCTG
IL-1β	Fw: AGCTACGAATCTCCGACCAC
	Rv: CGTTATCCCATGTGTCGAAGAA
TNF-α	Fw: AGCCCATGTTGTAGCAAACC
	Rv: TGAGGTACAGGCCCTCTGAT
MMP9	Fw: CACTGTCCACCCCTCAGAGC
	Rv: GCCAACTTGTCGGCGATAAGG
ANG2	Fw: CTCGAATACGATGACTCGGTG
	Rv: TCATTAGCCACTGAGTGTTGTTT

## Data Availability

Data is contained within the article and Supplementary Materials.

## Supplementary Materials

The following supporting information can be downloaded at: https://www.mdpi.com/article/10.3390/ijms25031774/s1.
