# Supplementary material for: Molecular Mechanisms and Therapeutic Implications of Human Pericyte-like Adipose-Derived Mesenchymal Stem Cells in an In Vitro Model of Diabetic Retinopathy
Source: Int J Mol Sci. 2024 Feb 1;25(3):1774. doi: 10.3390/ijms25031774 (PMC10855418; doi:10.3390/ijms25031774)
Supplement: Supplementary file 1 [file ijms-25-01774-s001.zip › ijms-2700861-supplementary.pdf]

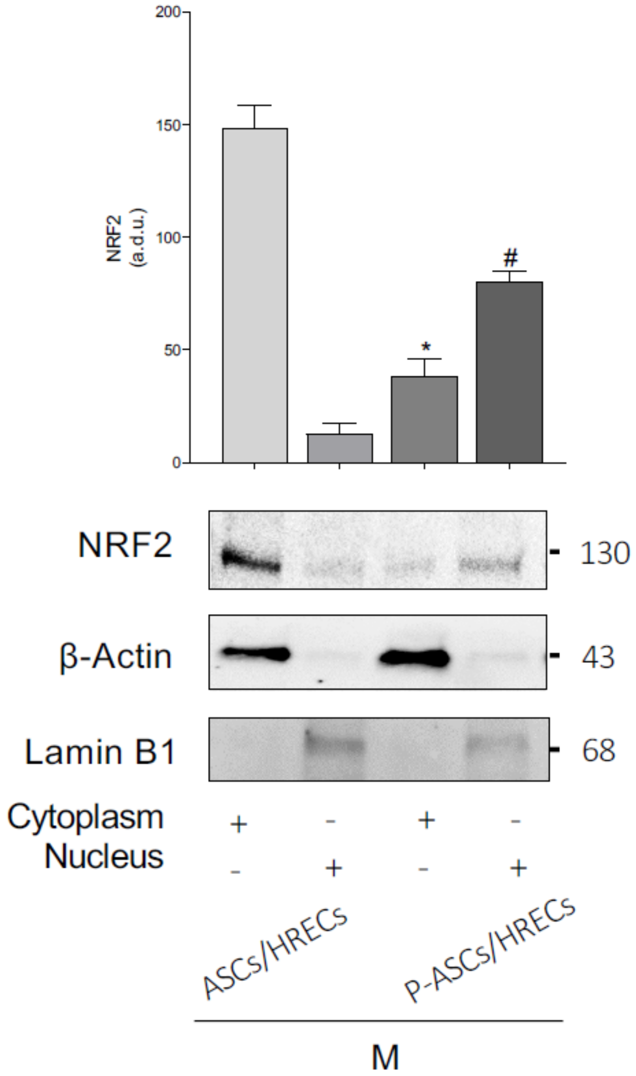

Figure S1. Evaluation of Nrf2 in the cytoplasm and nuclei of human adipose-derived mesenchymal stem cells (ASCs) and in human pericyte-like ASCs (P-ASCs) co-cultured with human retinal endothelial cells (HRECs) (indirect-cocultures). Data were gathered by Western blot analysis from samples that were cultured under 5 mM glucose plus 20 m MD-mannitol (M) as osmotic control. β-Actin and Lamin B1 were used to verify the equal loading of 30 µg protein per lane in cytoplasm and nuclei, respectively. Image J software was used to carry out densitometric analysis of the immunoblots, indicating protein quantification of each band (in arbitrary densitometry units, a.d.u.). Quantitative analysis of Nrf2 was normalized to β-Actin and Lamin B1. The bars represent means ± SD of three independent experiments performed in triplicate. Statistically significant differences, determined by one-way ANOVA, followed by Dunnett's multiple comparisons test, are indicated: * $p<0.05$ *vs.* cytoplasmic fraction of ASCs/HRECs in M; # $p<0.05$ *vs.* nuclear fraction of ASCs/HRECs in M.
